# MINDFuL: a Multi‐center, Randomized, Double‐blind, Placebo‐controlled Phase 2 Clinical Trial of XPro1595 for Patients with Early Alzheimer's Disease and Biomarkers of Inflammation

**DOI:** 10.1002/alz70861_108386

**Published:** 2025-12-23

**Authors:** Judy Jaeger, Sharon Cohen, Therese Blomberg, Parris Pope, Kim Staats, Sarah Barnum, Lisle Kingery, Genevieve Wills, Melanie Buitendyk, Tara Lehner, Malu Gámez Tansey, RJ Tesi, CJ Barnum

**Affiliations:** ^1^ CognitionMetrics, LLC, Stamford, CT USA; ^2^ Toronto Memory Program, toronto, ON Canada; ^3^ INmuneBio, Inc., Boca Raton, FL USA; ^4^ Consulting Neuropsychologist, Geneva, NY USA; ^5^ Allucent, Schiphol, Schiphol Netherlands; ^6^ Allucent, Saint‐Laurent, QC Canada; ^7^ Indiana University School of Medicine, Indianapolis, IN USA

## Abstract

**Background:**

Dysfunctional inflammatory/immune processes are recognized as key contributors to the pathogenesis and progression of Alzheimer's Disease (AD). Tumor necrosis factor (TNF) is an apex inflammatory/immune signaling molecule that drives a range of immune functions that include neurotoxic neuroinflammation via soluble TNF and homeostatic maintenance and repair of neurons via transmembrane TNF. XPro1595 was designed to selectively neutralize soluble TNF while preserving transmembrane TNF resulting in a polarization of immune cell function to a reparative and neuroprotective state.

**Method:**

MINDFuL (NCT05318976, EUCT2023‐505396‐71‐00) is a proof of concept, global, randomized, double‐blind, placebo‐controlled, Phase 2 clinical trial in participants with early AD and at least one biomarker of inflammation. Patients with biomarker confirmed mild cognitive impairment (MCI) due to AD or mild AD dementia (mAD) and biomarkers of inflammation were randomized 2:1 to receive either XPro1595 (1.0 mg/kg) or placebo, respectively, administered once‐per‐week by subcutaneous injection for 23‐weeks. The primary outcome is the change from baseline on the Early Mild Alzheimer’s Cognitive Composite (EMACC). Key secondary outcomes include change on the CDR‐SB, E‐Cog, and NPI‐12. Additional secondary outcomes include ADCS‐ADL, blood biomarkers of AD related pathology and neuroinflammation, and volumetric MRI.

**Result:**

MINDFuL enrolled 208 early AD patients, MCI (*n* =92) or mAD (*n* =116), 51% were female (*n* =106). All primary and secondary endpoints will be presented. The data will include cognitive and functional endpoints, a detailed safety analysis, blood biomarkers, and neuroimaging data.

**Conclusion:**

MINDFuL is a proof‐of‐concept study evaluating the efficacy and safety of XPro1595 (1.0 mg/kg/wk) in patients with early AD and biomarkers of inflammation. Detailed results of unblinded cognitive, functional, safety, and biomarker data will be presented.

